# Herpes Simplex Virus and Human Papillomavirus Coinfections in Hyperimmunoglobulin E Syndrome Presenting as a Conjunctival Mass Lesion

**DOI:** 10.1155/2017/1650841

**Published:** 2017-10-12

**Authors:** Mitra Akbari, Ramin Elmi

**Affiliations:** ^1^Eye Research Center, Guilan University of Medical Sciences, Rasht, Guilan, Iran; ^2^Legal Medicine Organization, Rasht, Guilan, Iran

## Abstract

Hyperimmunoglobulin E syndrome (HIES) or Job's syndrome is a rare immunodeficiency disease with less than 200 cases reported worldwide, among which few cases are reported with lesions due to herpes simplex virus (HSV) or human papillomavirus (HPV). This case study presents a rare case of HIES with coinfection of HSV and HPV. A 12-year-old boy, previously diagnosed with HIES, presented with a large conjunctival mass lesion. The presence of HPV in the lesion was confirmed by biopsy and by using the line-probe assay method to detect the HPV genome. However, the mass lesion did not respond to anti-HPV therapy with topical interferon-*α*2b (IFN-*α*2b) and oral cimetidine but improved promptly after intravenous (IV) acyclovir, which is often administered for cutaneous herpetic lesions. This suggested the presence of HSV in the conjunctival mass. Review of pathology and HSV immunohistochemical staining confirmed the presence of HSV as a coinfection. The likelihood that the mass arose from an abnormal host response to HSV and HPV due to HIES was considered, but coexisting infection with these two viruses and HIES has not been reported in the literature; therefore, such cases require further investigation.

## 1. Introduction

Hyperimmunoglobulin E syndrome (HIES) or Job's syndrome was first described by Davis et al. in 1966 [1] as a collection of rare immunodeficiency syndromes usually diagnosed in childhood based on a triad of elevated serum hyperimmunoglobulin E (IgE) levels, chronic dermatitis, recurrent pyogenic infections, or other clinical features that vary based on autosomal dominant or recessive disease [2]. Only 200 cases of HIES have been reported worldwide, identified using various molecular etiologies, such as a mutation in signal transducer and activator of transcription 3 (STAT3) and dedicator of cytokinesis 8 (DOCK8) genes [3].

Most cases of HIES are sporadic, although the disease can be inherited as an autosomal dominant or autosomal recessive trait. The basic immunologic defects of HIES are not clearly defined; however, abnormal neutrophil chemotaxis due to decreased production or secretion of interferon *γ* plays a primary role in the immunopathogenesis of this syndrome, and distorted T-helper (Th1/Th2) cytokine profiles contribute to impaired cellular immunity and specific patterns of infection susceptibility. Chronic refractory viral infection, particularly herpes simplex virus (HSV), molluscum contagiosum, varicella-zoster virus, and human papilloma virus (HPV) due to qualitative T-cell defects, can occur [4]. Presentations of HSV infections alongside this syndrome include large soft tissue masses around the nose, ears, and eyelids [5, 6].

In addition to the various unusual manifestations of HIES, HSV infection is observed as recurrent lesions with keratitis [2] and has been reported to present as a skin mass lesion as well [2, 5, 6]. In contrast, only two case reports have mentioned HPV with HIES [7, 8]. Here, a case of HIES with coinfections of HSV and HPV is reported, which presented as a conjunctival mass lesion. It is proposed that the patient's immunodeficiency may have accounted for the compromised response to the viruses.

## 2. Case Report

**Figure 1:**
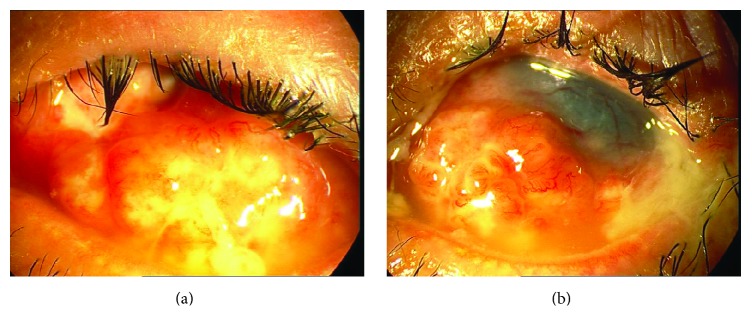
((a) and (b)) Photo-slit lamp images demonstrating the rapid development of a conjunctival mass lesion within a few weeks on the patient's left eye.

A 12-year-old boy presented to the Cornea Clinic of our hospital with a conjunctival mass lesion on his left eye, which had developed over the course of a few weeks. During a slit lamp examination, the lesion was found to be lobulated with corkscrew vessels that infiltrated the temporal, nasal, inferior and superior bulbar, and forniceal conjunctiva (Figures 1(a) and 1(b)). He had a positive history of recurrent herpetic keratitis on the same eye and had undergone penetrating keratoplasty six years earlier. However, his corneal graft had failed with total opacity and vascularization due to recurrent herpes simplex keratitis (HSK) in the corneal graft. The right eye was normal.

His history revealed that he had suffered from eczematous skin lesions, recurrent skin bursts, and sinopulmonary infections since early childhood and recurrent generalized HSV infection. He had been diagnosed with HIES at the age of three based on elevated serum IgE levels and other clinical features. He did not have a positive family history of HIES or any other immunodeficiency.

At the onset of the lesion discussed here, a differential diagnosis of a conjunctival lymphoid tumor, conjunctival papilloma, and ocular surface squamous neoplasia was given. Because the patient was prone to recurrent HSK, recurrent herpetic lesions were not considered in the differential diagnosis because the patient presented a papillomatous mass lesion of the conjunctiva. Thus, the pathogenesis was suspected to be HPV because the dendritic lesion and stromal infiltration had not be seen in the failed graft. For a specific diagnosis, an incisional biopsy was obtained from the conjunctival lesion, and sections were prepared and stained with hematoxylin and eosin using the periodic acid-Schiff technique to observe the sections under light microscopy (Olympus BX43, Tokyo, Japan). Microscopic examination revealed conjunctival tissue with dense, diffuse, acute, and chronic inflammatory cells that infiltrated the lamina propria and were rich in eosinophil. Eosinophilic conjunctivitis with pseudoepitheliomatous hyperplasia was the pathologic diagnosis. An examination of the specimens for the presence of HPV was requested, and genotyping was performed by a DNA analysis using a line-probe assay method that revealed genotype 52 (high-risk) HPV.

**Figure 2:**
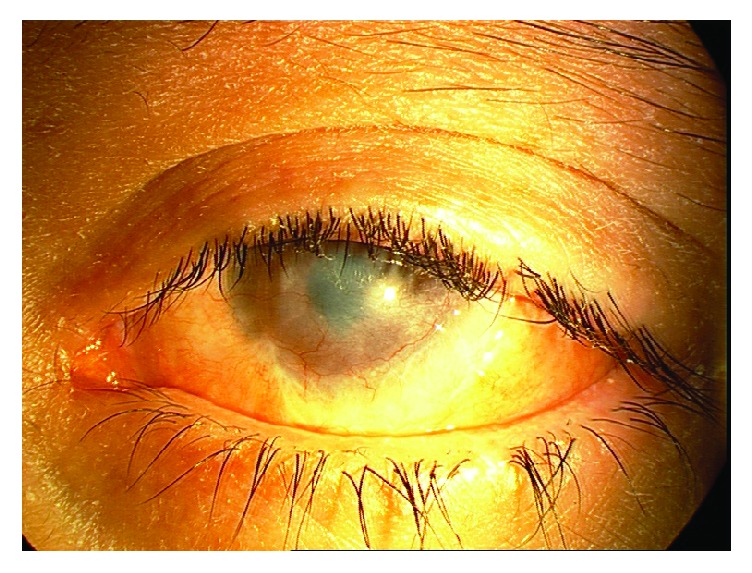
Photo-slit lamp image demonstrating rapid remission of the lesion after treatment with IV acyclovir in the patient's left eye.

The patient was treated with topical interferon-*α*2b (IFN-*α*2b) (3 MIU/cc of PDferon-B; Pooyesh Darou Co, Iran), 1,000,000 IU/mL, four times a day and oral cimetidine (Iran Daru, Iran, Tehran), 200 mg, four times a day for HPV and was given a low dose of oral acyclovir (400 mg/day) to prevent recurrence of HSK. There was no clinical response to this management at the three-month follow-up. In the next stage of treatment, the patient should have undergone excision and debulking of the mass lesion, but extensive herpetic lesions had recurred on the skin of the patient's head and neck. Therefore, the patient received intravenous (IV) acyclovir for systemic herpetic disease (600 mg/three times a day), prescribed by a pediatrician. The size of the conjunctival mass lesions decreased rapidly after this treatment, and the lesion completely resolved within a few days (Figure 2). Due to this positive response to IV acyclovir, the presence of HSV in the lesion was suspected and a reexamination of the pathology sample using immunohistochemical staining for HSV revealed the presence of this virus and proved coinfection.

## 3. Discussion

A rare case of known HIES is presented with a conjunctival mass lesion created by coinfection of HSV and HPV, which were suspected to be induced by the immunodeficiency syndrome that increased the patient's vulnerability to viral infections. In primary HIES, susceptibility to infections caused by bacteria, fungi, and viral agents increases and can create cutaneous infections of HSV, herpes zoster, molluscum contagiosum, and HPV [9]. The mechanism of immunodeficiency in patients with HIES is attributed to impairment of cytokines and abnormalities in T-lymphocyte function [10]. Combined immunodeficiency in this syndrome is explained by the failure of immunological synapse formation, leading to reduced natural killer cell cytotoxicity and insufficient specific immunity development with a lack of T- and B-memory cells [5]. In this case study, the classic manifestations led to an initial diagnosis of HIES at the age of three that is postulated to be responsible for susceptibility to an unusual coinfection of HSV and HPV.

The patient manifested a papillomatous mass lesion, which was suspected to be an HPV infection. In childhood, papilloma represents 7–10% of conjunctival tumors and possibly plays a role in HPV [11]. The management of papilloma is difficult because local excisions through cryotherapy may not excise diffuse and multifocal lesions; thus, multiple topical treatments have been suggested, including interferon-alpha (IFN-*α*) and mitomycin-C, with variable success rates [12, 13]. Several adjuvant therapies have also been suggested, such as oral cimetidine, which acts as a systemic immunomodulatory [14]. Accordingly, the current patient was treated with sufficient treatments for HPV, including topical IFN-*α*2b and oral cimetidine, but there was no clinical response to anti-HPV management at the three-month follow-up, although the lesion resolved a few days after administration of 600 mg/three times a day of IV acyclovir. It was concluded that HSV was the main pathogenic agent responsible for lesion formation with a coinfection of HPV, which misled the management process.

A similar case was reported in the literature of a tumorous HSV blepharoconjunctivitis in a patient with a DOCK8 deficiency. She developed progressive blepharoconjunctivitis of the left upper and lower eyelid. In this case, after the initial IFN-*α* injections, rapid improvement in the lesion occurred, while pretreatment with systemic acyclovir and oral methylprednisolone had no effect on the lesion [5]. This study was similar to the current study, based on the presence of HSV in the tumor-like lesion associated with HIES; however, its findings were contrary to the current findings, which found that use of intravenous acyclovir significantly impacted the resolution of the lesion. The previous study did not define the dose of acyclovir, and the concomitant use of high-dose systemic steroids may have disrupted the effect of this antiviral agent. In addition, no effect on the lesion was observed in the current study after the use of topical IFN-*α*; however, systemic IFN-*α* may be more useful than topical IFN-*α*.

Another case of an unusual pathogenicity of the HSV in HIES was reported in a seven-year-old girl who presented with large soft masses rising from her nostril and from behind her ear. The presence of HSV within these lesions was confirmed. A mild decrease in CD_4_ cells was proposed as an immunological mechanism for this unique manifestation of HSV coinfection with HIES. The mass lesions improved promptly under antiviral therapy with acyclovir [6], which agrees with the results of the current study.

HPV-6 and HPV-11 are major HPV types that are responsible for conjunctival lesions [15]. In the current patient, genotype 52 revealed high-risk (for cancer) HPV, which suggested the severity of HPV infection in HIES is high. Few reports are available in this regard [7, 8], and further research and clinical suspicion are both required for the proper diagnosis of such coinfections in HIES.

Here, an immunological mechanism is proposed for a unique manifestation of coinfection of HPV and HSV in HIES, which has not previously been presented in the literature. No treatment modality is curative for the basic defects of HIES, in terms of cytokine/chemokine derangement, but it is hoped that bone marrow transplants and monoclonal anti-IgE (omalizumab) would be successful future treatments [4]. IFN-*α* treatment was found to be beneficial in the treatment of virally triggered tumors; thus, continuous IFN-*α* treatment is an option for patients who are ineligible for bone marrow transplantation [5]. The role of IV acyclovir in the treatment of HSV-induced lesions has been clearly shown in both this case and previous cases in the literature.

Identification of coinfections presented as conjunctival mass lesions in HIES is rare and diagnostically challenging. Such infections should be distinguished from other possibilities, such as primary HPV infection and other tumoral lesions, to ensure appropriate treatment and to avoid unnecessary and ineffective interventions, such as debulking of the lesion. The presence of these viruses in this syndrome, especially in the form of mass lesions, should always be kept in mind.

## 4. Conclusion

To the best of our knowledge, this is the first description of an unusual and coexisting HSV and HPV related conjunctival mass lesion in a patient with HIES that was resolved promptly after treatment with intravenous acyclovir.
